# Nonepileptic, Stereotypical, and Intermittent Symptoms After Subdural Hematoma Evacuation

**DOI:** 10.7759/cureus.18361

**Published:** 2021-09-28

**Authors:** Varun Jain, William Remley, Arvind Mohan, Emma L Leone, Srishti Taneja, Katharina Busl, Leonardo Almeida

**Affiliations:** 1 Neurology, University of Florida, Gainesville, USA; 2 Neurology, Lake Erie College of Osteopathic Medicine, Jacksonville, USA; 3 Neurosurgery, University of Florida, Gainesville, USA; 4 Neurology, Avalon University School of Medicine, Youngstown, USA; 5 Neurocritical Care, University of Florida, Gainesville, USA

**Keywords:** subdural, seizure, nesis, nonepileptic, cortical, spreading, depolarization, intermittent, stereotypical

## Abstract

Transient neurological deficits can occur in the setting of subdural hemorrhages with subsequent unremarkable electrodiagnostic and radiological evaluation. This scenario is rare and can be difficult for physicians to interpret. These transient neurological deficits are thought to result from relative ischemia, secondary to a lesser-known concept known as cortical spreading depolarization. These transient neurological deficits are thought to result from relative ischemia, secondary to a lesser-known concept known as cortical spreading depolarization, which may present clinically as nonepileptic, stereotypical, and intermittent symptoms (NESIS). In these instances, patients are often misdiagnosed as epileptics and committed to long-term antiseizure drugs. We present a 51-year-old patient developing acute global aphasia following the evacuation of a subdural hematoma, with no significant findings on laboratory, microbiological, electrodiagnostic, or radiological evaluation. The patient experienced spontaneous improvement and returned to baseline in the subsequent weeks. Increased awareness of NESIS as a cortical spreading depolarization phenomenon can improve patient care and prevent both unnecessary, extended medical evaluations and therapeutic trials.

## Introduction

Acute and chronic subdural hematomas (SDH) are common reasons for neuro-intensive care unit admissions. As populations have aged, the incidence of combined acute and chronic SDH has also increased from 1.7/100,000/year in 1975 to 20.6/100,000/year in 2011 [1-3]. While the most common cause of SDH is head trauma, there are other factors that contribute to the incidence of SDH such as antithrombotic agents, anticoagulants, cerebral atrophy, cerebral aneurysms, and intracranial hypotension [4,5]. Of all head traumas from mild to severe, an average of 11% is complicated by an acute SDH [4,6]. Patients with an acute SDH present with symptoms ranging from their neurological baselines to varying levels of consciousness to coma [7]. Interestingly, focal deficits may be ipsilateral or contralateral to the side of the SDH [8]. In some cases, elevated intracranial pressure and mass effect can lead to cerebral hypoperfusion and infarction [9]. SDH commonly cause seizures, with an incidence of 28% in patients with acute SDH and 10% in patients with chronic SDH [10]. Also, there is a mixed time course of seizure presentation, with a pre-operative incidence of 16% and a post-operative incidence of 24% [10].

Rarely, patients who undergo SDH evacuation can post-operatively develop transient neurological deficits with no correlation on surface electroencephalogram (EEG) and no evidence of radiological worsening on repeat imaging [11]. This phenomenon is thought to arise from cortical spreading depolarization, which is thought to lead to transient relative neuronal ischemia [12]. Symptoms resulting from this etiology may carry different prognostic risk factors and may require treatment with medications targeting entirely different mechanisms of action [12-14]. There is a need for different diagnostic techniques, such as electrocorticography, and for research that further explores delayed neuronal injury from blood products to further elucidate and assist with early recognition of this phenomenon, which will allow clinicians to provide better care for these patients.

## Case presentation

A 51-year-old Caucasian male with a past medical history of hypertension and chronic obstructive pulmonary disease presented to the emergency room for evaluation of headaches for three days following a mechanical fall resulting in hitting his head. The left-sided headache was 8/10 severity on the visual analog scale and was dull and constant. The headache was not associated with nausea, vomiting, and was not sensitive to light or sound. He received minimal pain relief from Excedrin and ibuprofen. He denied any weakness, numbness, vision, or speech changes.

**Figure 1:**
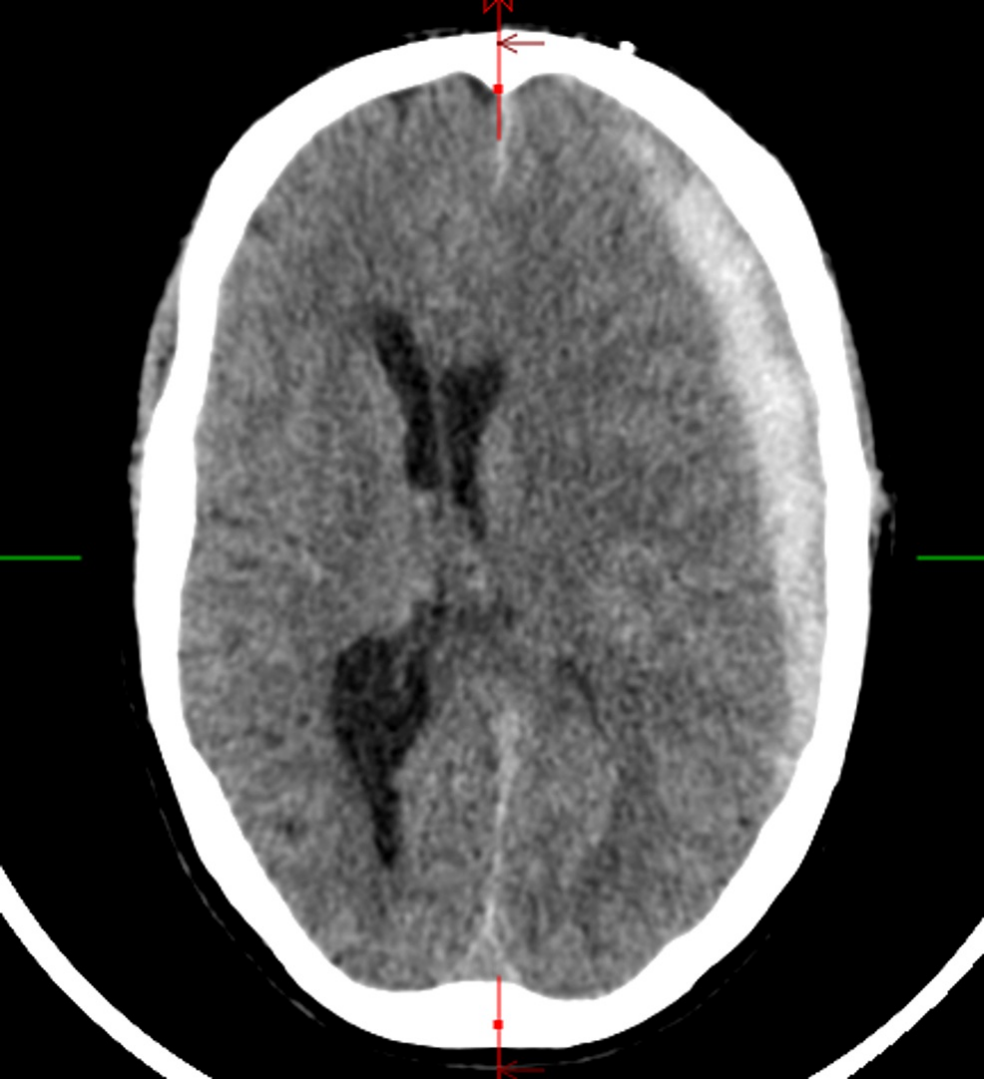
Noncontrast axial head CT at admission showing SDH. SDH = subdural hematoma

On the initial evaluation, the patient was afebrile, blood pressure was 115/82 mmHg, pulse was 85 beats/minute, respiration rate was 12/min, and oxygen saturation was 100% on room air. His Glasgow Coma Scale was 15, alert and oriented to person, place, and time. He could perform simple calculations. The speech was fluent with normal naming, repetition, reading, writing, and comprehension. Examination of cranial nerves, motor and sensory functions were intact. Coordination and gait were normal. A noncontrast computerized tomography (CT) scan of the head demonstrated an acute left SDH causing significant mass effect and midline shift (Figure 1).

**Figure 2:**
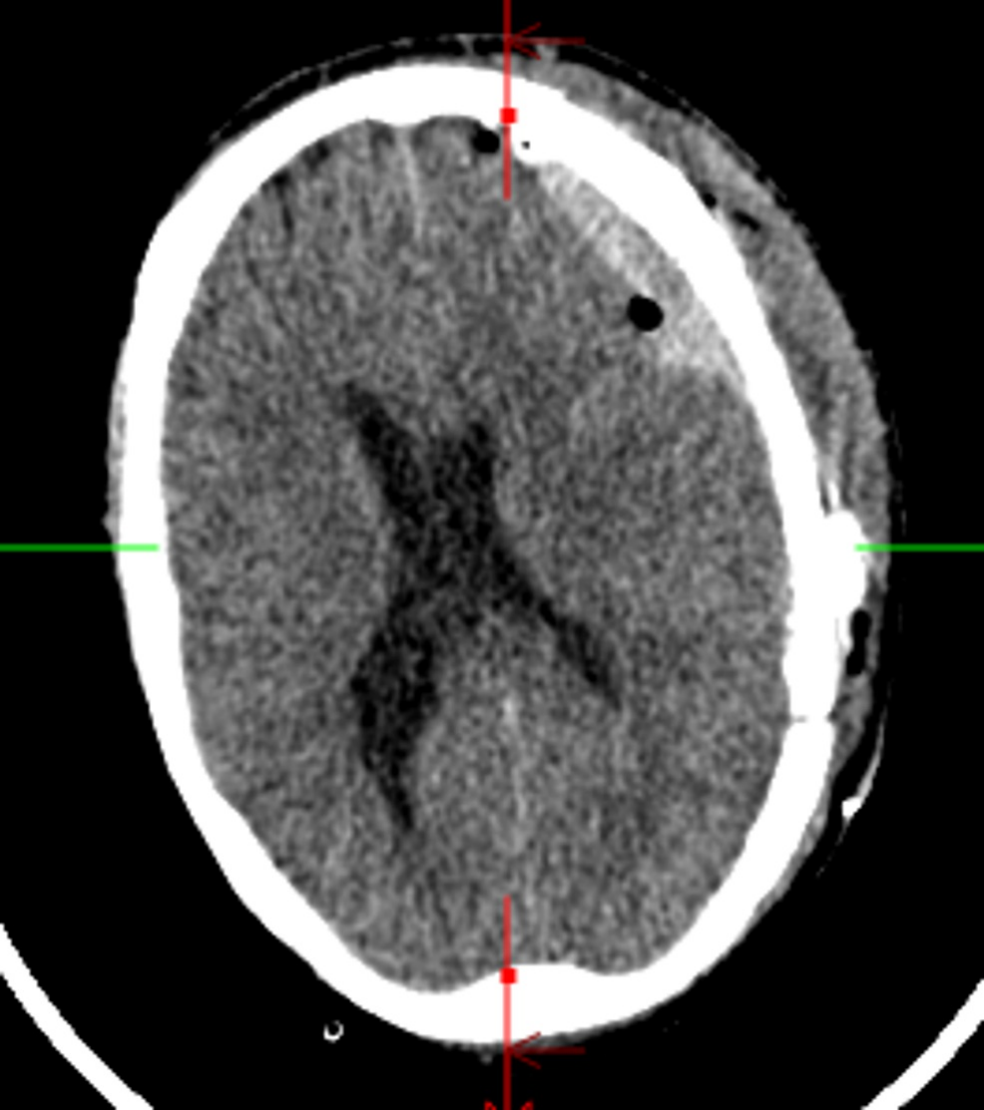
Noncontrast axial head computed tomography (CT) after decline in examination showing SDH without interval worsening. SDH = subdural hematoma

After the CT scan was discussed with the neurosurgeon, he was emergently taken to the operating room for evacuation of the left SDH, which was performed unremarkably. A standard craniotomy was performed, and the cortex was unremarkable. The physical examination after the procedure was unchanged. On post-operative days zero and one, he was alert and oriented to person, place, and time. He had normal speech and was able to follow commands. On post-operative day two, he acutely developed confusion and mixed aphasia. The patient could only speak “ya, ya” to all questions. He moved all four extremities anti-gravity and did not follow commands. To evaluate his acute change, a stat repeat CT head and long-term EEG were obtained (Figure 2). CT head showed overall improvement and no new blood reaccumulation, with a decrease in mass effect and midline shift, as well as post-surgical changes.

Two hours after his acute worsening of symptoms, his symptoms resolved and he was back at his baseline, alert and oriented to person, place, and time with normal language and followed commands. Two hours after he was back at his baseline, he again became confused and could not follow commands, but he was now mute. A long-term surface video EEG monitoring was conducted for 72 hours, which failed to capture any epileptiform discharges or seizures. It showed background activity of predominantly delta waves with an admixture of theta and beta without an anterior-posterior gradient or a discernible posterior dominant rhythm at normal voltage. It showed frequent, unilateral, left temporal rhythmic delta activity without associated plus features such as rhythmic or fast sharp activity. His complete blood count, basic metabolic panel, ammonia, liver enzymes, thyroid function tests, urinalysis, urine and blood cultures, and urine drug screen were all unremarkable. MRI was attempted twice to exclude ischemia, but could not be completed due to agitation, then hypotension related to Precedex administration. He remained on an empiric trial of levetiracetam for seven days. There were no new medications or adjustment of chronic medications that could have contributed to his neurological changes. An empirical trial of antipsychotics to evaluate for delirium did not improve the symptoms.

Over the subsequent days, he experienced limited improvement. On post-operative day eight, he was able to name objects and follow simple commands, although he still had impaired repetition. He was able to speak short sentences, such as, “I’ll be fine” and “give it a day or two”. He was still having phonemic paraphasic errors, phonemic jargon, difficulty with complex commands, reading deficits, and word-finding difficulties. He was discharged to home with home services. At his one-month follow-up, he was found to have significant improvement in his language skills and at the two-month follow-up, he was at his neurological baseline.

## Discussion

Our 51-year-old patient who underwent SDH evacuation was at his neurological baseline on post-operative days zero and one, then on post-operative day two, he developed acute onset confusion, inability to follow commands, and mixed aphasia that progressed to mutism. CT head and continuous 72-hour video EEG were performed to evaluate for seizure and the reaccumulation or worsening of SDH, and both were unremarkable. His symptoms did not significantly improve with trials of anti-epileptic or anti-psychotic medications.

Global aphasia without hemiparesis is often due to an ischemic event or seizure [15,16]. Although a limiting factor of our case report is that an MRI was unable to be successfully performed, our patient’s development of global aphasia without hemiparesis is unlikely to have resulted from an acute ischemic event based on a lack of corresponding CT head findings [15,17]. CT head was performed immediately after he developed symptoms and 48 hours after the onset of symptoms, and neither showed hypodense areas suggestive of infarction. For an ischemic event to cause global aphasia, the volume of the stroke is expected to be large enough that the hypodensity should be visible on the CT head in the frontal or temporal lobes [18,19]. Large ischemic events that cause global aphasia typically also cause contralateral hemiparesis [15]. In a case study of four patients with global aphasia without hemiparesis, each patient had hypodensities on CT [15]. A distinct difference between our patient and each of these four patients with global aphasia without hemiparesis is that these four patients had modest improvements in their speech that did not return to their baseline [15]. Of the four patients with global aphasia, two improved to transcortical sensory aphasia, one to a Broca’s aphasia, and one to a Wernicke’s aphasia [15]. Additionally, because our patient was at high risk for seizures given his craniotomy for SDH evacuation, we performed an EEG to rule out seizures of which did not exhibit epileptiform activity [20]. To address this possible etiology, our patient was started on anti-epileptics that were discontinued after one week due to a lack of marked improvement in our patient’s symptoms, suggesting an alternative etiology [21]. EEG findings in NESIS must be nonspecific, such as a normal EEG, diffuse slowing, or other nonspecific findings [11].

Following SDH evacuation, some patients have transient neurological symptoms without epileptic findings on electroencephalography and without significant interval findings on repeated radiological evaluation [22,23]. The etiology of such changes is yet to be fully elucidated, but it has been theorized that these transient focal deficits occur due to cortical spreading depolarization or delayed neuronal injury from blood products, although the data and research to support these theories are still developing [11,24]. Spreading depolarizations have been measured with a high degree of accuracy and interobserver reliability by subdural platinum electrodes that produce direct, current-coupled recordings that can be used to identify and assess the impact of spreading depolarizations [25]. Electrophysiological evidence of these recordings have been abundantly found in individuals with pathologies such as aneurysmal subarachnoid hemorrhage, delayed ischemic stroke after subarachnoid hemorrhage, malignant hemispheric stroke, spontaneous intracerebral hemorrhage, and traumatic brain injuries [26].

In 2019, single-center retrospective study of 59 SDH patients by Driver et al. showed that SDH patients who had transient neurological symptoms without correlation on EEG were more likely to have aphasia, dysphasia, and prolonged episodes of symptoms [27]. In contrast, clonic movements, impaired awareness, positive symptomatology, and response to epileptic drugs were associated with positive EEG findings [27]. Given our patient’s persistent symptoms, lack of correlation between symptoms and EEG, and lack of response to levetiracetam, we suspected that a seizure was not likely the etiology [27]. Levesque et al. postulated that cortical spreading depolarization may be the etiology behind this presentation and coined the term nonepileptic, stereotypical, and intermittent symptoms (NESIS) to define this syndrome [11,28].

Cortical spreading depolarization (CSD) is a depolarizing wave spreading at a slow velocity (2-5 mm/min) in cerebral gray matter followed by electrical silence, typically lasting 5-20 minutes, followed by complete resolution of symptoms [14,29]. This increases the metabolic demand which perfusion cannot meet, leading to relative ischemia [26,30]. The incidence of CSD in chronic SDH is about 26%, 60% in TBI, 80% in SAH, and 90% in large hemispheric stroke, and CSD’s energy demand is estimated to be four to eight times that of a focal seizure [31]. Cortical spreading depolarizations in the cortex translate into slow potential changes and depression on scalp EEG [32].

Optimal data and scientific research on CSD in SDH are lacking. The papers previously published by Moster et al. in 1983 described 15 patients with SDH who had transient neurological deficits, of which nine patients had aphasia [33]. In 1992, Kaminski et al. published a review of 35 SDH patients who had transient neurological deficits, with aphasia occurring most commonly, in 77% of patients [34]. A limiting factor of this article was that only seven patients had an EEG performed. 

In 2017, Alkhachroum et al. described a case of transient left hemibody weakness status-post evacuation of SDH with evidence of a 10-second suppression of all frequencies correlating with a syncopal event that immediately preceded the onset of neurological symptoms [35]. Transcranial doppler in the patient showed low mean flow velocities in the right MCA, and the MRI was negative for acute diffusion restriction [35].

The expected time course of symptom resolution in NESIS is still being elucidated [11]. Case studies of patients with SDH have reported episodes of transient neurological deficits that lasted from 5 minutes to 24 hours [34,36]. In a study of 15 patients with transient neurologic deficits with a concomitant SDH, these episodes continued to occur for up to 90 days and occurred for an average of 21 days [33]. Given that cortical spreading depolarization has been shown to cause an electrical silence for up to 20 minutes, our patient’s symptoms are unable to be sufficiently explained by this concept [29]. The lack of other explanations for our patient’s symptoms makes this case report a contribution to the growing NESIS literature, as our patient’s presentation is thought to be most consistent with a diagnosis of NESIS [11]. Our patient had a prolonged episode of aphasia and dysphagia with a slow recovery without correlating epileptic discharges on long-term surface EEG or correlating radiological evidence. He did not have any positive symptoms (delusions or hallucinations), clonic movements, or a response to levetiracetam. Unfortunately, our suspected diagnosis of NESIS could not be further supported as our institution could not perform electrocorticography. The diagnosis of NESIS was made and his antiseizure drugs were not continued beyond week one. At his follow-up visit one month later, his examination showed a return to baseline with normal language skills, which was again strong evidence for our diagnosis of NESIS.

## Conclusions

Transient neurological deficits after SDH evacuation can be especially enigmatic and stressful for both patients and physicians when there is an absence of objective findings on both imaging and long-term surface electroencephalography. Knowledge of CSD and NESIS can help to make diagnoses in such cases, thereby preventing patients from unnecessary antiseizure drugs. NESIS is a growing term and syndrome, which not only imbibes the concept of CSD but also helps to characterize its symptoms. Because the data and research regarding CSD in SDH are scarce, larger studies are needed to evaluate the incidence and prognosis, confirm the etiology, and guide the management of this syndrome.
